# Efficacy of exergaming dance and aerobic dance on young adults’ enjoyment, situational motivation, self-efficacy, and steps

**DOI:** 10.3389/fpsyg.2025.1573954

**Published:** 2025-05-01

**Authors:** Kai Tan, Anna Kate Autry, John Oginni, Kun Tao, Zan Gao

**Affiliations:** ^1^College of Kinesiology and Health Science, Huaihua University, Huaihua, China; ^2^Webb School, Knoxville, TN, United States; ^3^Department of Kinesiology, Recreation, and Sport Studies, University of Tennessee, Knoxville, TN, United States

**Keywords:** active video game, intrinsic motivation, physical activity, self-efficacy, self-determined motivation

## Abstract

**Introduction:**

This study explored differences in young adults’ enjoyment, situational motivation, self-efficacy, and physical activity between two content-identical exercise formats: exergaming aerobic dance and traditional aerobic dance.

**Methods:**

A total of 40 young adults (20 females; Mage = 20.38) from a Chinese university participated in two separate 12-min dance sessions: (1) a non-stop exergaming aerobic dance using the Xbox 360, *Kinect Just Dance* and (2) a traditional aerobic dance led by an experienced instructor. Psychological measures of self-efficacy, enjoyment, and situational motivation (including intrinsic motivation, identified regulation, external regulation, and amotivation) were assessed using validated questionnaires after each session. Physical activity levels were measured using a research-grade pedometer to track steps. A repeated-measures MANOVA compared the outcomes between the two dance modalities.

**Results:**

Statistically significant differences were observed between the two dance sessions for the overall model (*p* < 0.05). Participants reported higher enjoyment during the exergaming dance session compared to the traditional aerobic dance [*F* (1,39) = 3.59, *p* = 0.05, η^2^ = 0.07]. Intrinsic motivation were significantly higher for exergaming dance than for the traditional format [*F* (1,39) = 3.83, *p* < 0.05, η^2^ = 0.09]. However, participants achieved significantly more steps per minute in the traditional aerobic dance compared to the exergaming session [*F* (1,39) = 39.79, *p* < 0.01, η^2^ = 0.51]. No other significant differences were found for the remaining outcomes.

**Discussion:**

These findings suggest that exergaming dance may enhance perceived enjoyment and intrinsic motivation, leading to more time spent on exercise or physical activity, though it results in fewer steps per minute than traditional aerobic dance. These results are practically relevant for promoting long-term physical activity through game-like exercises, as higher enjoyment and motivation play a crucial role in maintaining physical activity.

## Introduction

The morbidity and mortality rates of chronic diseases, such as cardiovascular disease, diabetes, and obesity, are on the rise (Duncan et al., 2023), and physical activity (PA) is recognized as a key tool to counteract this trend (Jin et al., 2024; Ding et al., 2024). For example, research evidence has supported an inverse relationship between step counts and mortality rates from cardiovascular diseases (Sheng et al., 2021). The Centers for Disease Control and Prevention (CDC) (CDC, 2023) recommend engaging in 150 min of moderate-to-vigorous PA a week, including 2 days of muscle-strengthening activity per week.

Despite these being modest goals and seemingly achievable guidelines, many young adults - including college students-fail to meet them due to lifestyle choices, such as lack of time and limited access to PA equipment and facilities (Tarp et al., 2024; Calestine et al., 2017). Researchers have observed a significant rise in physical inactivity among children and young adults aged 9 to 22 years (Bennie et al., 2023). For instance, a survey of 222,159 Chinese college students revealed particularly low levels of PA among those aged 18 to 21 (Wang et al., 2017), even though PA has been shown to yield many positive health outcomes, including improved physical fitness, weight reduction, and enhanced psychological and mental well-being in the form of boosted self-esteem, motivation, and emotional balance (Ekelund et al., 2024; Bao et al., 2020). Unfortunately, PA participation remains low in China and many other regions, despite its well-documented benefits. Therefore, developing innovative and engaging strategies to encourage PA among young adults is essential to address this growing challenge.

Given the increasing number of video game users among college students (Przybylski et al., 2017), exergaming presents a promising solution by integrating PA into a familiar and engaging format.

Exergaming, or active video games (AVG) that require physical engagement (Baysden et al., 2021; Sousa et al., 2022; Comeras-Chueca et al., 2021), has proven effective in promoting PA among children and young adults (Pasco and Roure, 2022; Ye et al., 2019; Gao and Xiang, 2014; Gao et al., 2012). Exergaming involves video games that integrate physical movement into gameplay (Wang et al., 2017) and has demonstrated considerable potential for encouraging PA across various populations (Zhu, n.d.; Barnett et al., 2011; Edwards et al., 2017; Zeng et al., 2017). In the US, 70% of males and 50% of females aged 18 to 29 reported regularly playing video games (Brown, 2017). One of the most popular gaming platforms, Microsoft’s Xbox (Inlingo, 2024) (Microsoft, Redmond, WA, USA), achieved global sales of 7.5 million units in2023 (Ahmed, 2024). With the growing interest in video gaming among young adults, exergaming offers a promising approach to fostering PA and improving both psychological and mental well-being, particularly in college student populations.

## Literature summary

Studies have identified several psychological and mental benefits resulting from exergaming (Gao et al., 2019; Andrade et al., 2019; Lee et al., 2017) by revealing positive influences of exergaming on enjoyment, motivation, self-efficacy, social interactions, and self-esteem (Gao et al., 2014; McDonough et al., 2021; Zhang et al., 2015; Gao et al., 2011; Gao et al., 2013). A study by Krause and Jenny (2023) reported positive self-efficacy beliefs when exergaming is incorporated into physical education, while a correlation analysis also revealed that self-efficacy, enjoyment, and rate of perceived exertion (RPE) were positively associated, implying that competence in playing exergames may be linked to greater enjoyment of the gameplay and increased effort during participation (Zhang et al., 2015). Overall, research evidence has shown that exergaming can enhance motivation, self-efficacy, and enjoyment in college students (Sousa et al., 2022; Pasco and Roure, 2022; Gonçalves et al., 2021; McDonough et al., 2018, 2020; Liu et al., 2019).

The use of exergaming as a health promotion tool shows great promise for enhancing college students’ motivation to engage in PA (Gao et al., 2014). Research on college students’ PA motivation has highlighted varying levels of self-determined motivation in participation (Kilpatrick et al., 2005; Ntoumanis, 2001; Ryan and Deci, 2000; Ntoumanis, 2005; Zhao et al., 2024; Gu et al., 2025). According to Self-Determination Theory (SDT) (Deci and Ryan, 2000), four key factors influence the initiation and regulation of behavior: (a) intrinsic motivation (IM), involving activities pursued for inherent enjoyment or satisfaction; (b) identified regulation (IR), where individuals undertake activities they perceive as aligned with their personal goals and values; (c) external regulation (ER), driven by the pursuit of external rewards or avoidance of punishment; and (d) amotivation (AM), characterized by a lack of intention and minimal motivation. IM and IR reflect higher levels of self-determined motivation, often resulting in positive outcomes, whereas ER and AM represent lower motivation levels, which are associated with negative consequences (Ning et al., 2015). In simple terms, students are more likely to engage in and sustain an activity when they are intrinsically motivated - doing it for their own enjoyment or personal satisfaction (Guay et al., 2000). It is worth noting that while the Situational Motivation Scale—which captures the motivation individuals experience during a specific activity—may appear distinct from the broader framework of Self-Determination Theory (SDT), it is in fact firmly grounded in SDT’s principles. When implementing a new PA intervention, evaluating motivation is crucial. While IM is well-documented to support regular PA participation, it remains unclear whether a technology-based exercise modality would influence both situational motivation for PA and adherence to it.

Additionally, exergaming provides an engaging and enjoyable way for students to achieve the recommended levels of PA. Gao et al. (2020) conducted a systematic review to evaluate the impact of AVG on body composition and PA, confirming its positive effects in reducing body fat and increasing PA participation. Gao et al. (2015) further quantitatively examined the effects of exergaming on promoting PA, showing that AVG could serve as an alternative to sedentary behavior, though it may not suffice as a replacement for traditional PA and sports. However, further development and customization of specific games are necessary to mitigate potential threats and weaknesses associated with exergaming misuse (Benzing and Schmidt, 2018). One notable strength of exergaming is its appeal across different age groups, with college students showing significant interest in various exergaming approaches (Pasco and Roure, 2022). Moreover, researchers (Douris et al., 2012; Sadeghi and Jehu, 2022) found similar results, favoring exergaming over traditional aerobic exercise.

In summary, the literature highlights that different PA modalities of the same exercise may have varying effects on individuals’ enjoyment, situational motivation, self-efficacy, and PA levels.

However, few studies have yet compared the effects of exergaming aerobic dance versus traditional aerobic dance on various outcomes among college students (McDonough et al., 2018, 2020; Gao et al., 2013). While researchers have examined the effects of exergaming and instructor-led aerobic training (Hwang et al., 2023), there is still a lack of understanding regarding how exergaming dance directly compares to a traditional dance workout, particularly in terms of physical activity volume (steps) and motivation. This study aims to examine differences in young adults’ situational motivation, PA steps, self-efficacy, and enjoyment across these two modalities (exergaming aerobic dance vs. traditional aerobic dance led by an instructor).

Based on previous studies (Lee et al., 2017; Gao et al., 2015), we hypothesize that young adults will report higher enjoyment, IM, IR, and self-efficacy with exergaming aerobic dance. Additionally, we hypothesize that step counts will be similar in both dance sessions. Identifying and understanding the implications of this study could help categorize and determine specific effective exergaming programs for motivating engagement and promoting PA participation. Furthermore, the findings may provide health professionals and educators with innovative activities to encourage PA participation.

## Materials and methods

### Participants

A total of 40 young adults (20 females; Mage = 20.38, SD = 0.74) were recruited from a regional university in Southcentral China, with data collection conducted during the summer of 2018. The inclusion criteria for participation were: (1) enrollment at the university; (2) age between 18 and 25 years; (3) being in good health (i.e., absence of physical or mental conditions that could prevent participation in PA); and (4) provision of informed consent. To detect a mean difference in the primary outcome (step count) between the two dance conditions with 80% statistical power and a 5% significance level, and assuming a standard deviation of 200 steps per session, a total of 40 participants is required for this study. Participants’ recruitment started on July 1, 2018 and ended on July 7, 2018. Ethical approval from the university’s research ethics committee (IRB ID: No.20180509P10) was obtained, along with written consent from all participants, before initiating data collection.

### Procedures

Before participating in the dance sessions, trained research assistants collected participants’ demographic and anthropometric information. Participants completed two separate 12-min dance sessions—exergaming dance and instructor-led aerobic dance—administered in a counterbalanced order to control for potential order effects. In this design, participants were randomly assigned to begin with either the exergaming or the traditional dance session, followed by the other session after a rest period. This approach ensures that any differences in outcomes (e.g., motivation or PA levels) are not biased by the order in which the sessions were experienced, thereby enhancing the internal validity of the study. In brief, participants completed two distinct 12-min dance sessions in a campus fitness lab at the School of Health and Physical Education during pre-scheduled time slots: (1) a non-stop exergaming aerobic dance session using Xbox 360 Kinect’s *Just Dance – Just Sweat Around the World*; and (2) a traditional aerobic dance session led by an experienced instructor. To minimize potential carryover effects between sessions, participants were: (1) randomly assigned in a counterbalanced order, and (2) provided with a 10-min rest period between each cycling session to allow blood pressure to return to baseline, consistent with procedures used in previous research (Gonçalves et al., 2021; McDonough et al., 2020; Liu et al., 2019). During the rest period following each session participants completed questionnaires assessing enjoyment, situational motivation, and self-efficacy.

### Experimental conditions

For the exergaming session, an Xbox 360 Kinect console station was set up, featuring *Just Dance — Just Sweat Around the World* game. This dance/rhythm game requires players to mimic the movements of an on-screen dancer, combining PA with dynamic, interactive gameplay. The game offers a variety of high-energy dance routines set to international music tracks, providing a diverse and engaging workout experience. The game console was pre-set to eliminate transition time between dances, ensuring a continuous and vigorous activity flow. The system also provided visual feedback and performance scores, adding an element of gamification to enhance motivation and enjoyment during the session.

The instructor-led aerobic dance session, conducted on the opposite side of the fitness lab, was led by a certified and experienced instructor who guided participants through a structured series of aerobic dance routines designed to improve cardiovascular fitness and overall coordination.

This session consisted of a standardized aerobic dance routine designed to match the intensity level of the exergaming dance session. However, it featured different choreography and music selections, distinct from those used in the Just Dance game. The routine was instructor-led and developed to provide a comparable cardiovascular challenge, while indicating that the movement patterns and musical cues might not replicate the specific content or style of the exergaming platform. In detail, the dance instructor incorporated a variety of choreographed movements synchronized to upbeat music, progressing from a warm-up to more intensive dance sequences before concluding with a cool-down period. Each routine was tailored to accommodate varying fitness levels, ensuring inclusivity while maintaining a challenging and enjoyable atmosphere. Verbal cues and demonstrations enhanced participant engagement and motivation throughout the session.

### Measures

#### Demographic and anthropometric data

Participants self-reported demographic information, including their age, sex, ethnicity, and previous Just Dance experience through a structured questionnaire administered by trained research assistants. Anthropometric measurements were collected using validated instruments to ensure accuracy. Height was measured to the nearest centimeter using a stadiometer, while weight was recorded to the nearest 0.1 kilogram using a digital scale. These measurements were then used to calculate each participant’s Body Mass Index (BMI) using the formula BMI = weight (kg) / height (m^2^), providing a standardized measure of body composition for all participants.

#### Enjoyment and self-efficacy

Following each exercise session, participants completed a self-efficacy survey consisting of three items and an enjoyment survey with five items adopted from Zeng et al. (2017), and both measured on a 5-point Likert-type scale (1: strongly disagree to 5: strongly agree) (Zeng et al., 2017). The self-efficacy survey began with the stem question, “With regard to the [traditional aerobic dance or exergaming dance], I have confidence in…” Participants then rated their confidence in performing well, learning skills, and succeeding in the activity. The enjoyment survey assessed participants’ fun during the activity, preference for it over other activities, desire to engage in it more, preference for watching rather than playing (reverse-coded), and overall enjoyment. The mean scores from both surveys were calculated and used as indicators of self-efficacy and enjoyment for each type of exercise.

#### Situational motivation

A validated 16-item situational motivation (SM) survey (Guay et al., 2000), utilizing a 7-point Likert-type scale (1 = strongly disagree; 7 = strongly agree), was employed to assess participants’ SM. The survey, grounded in Self-Determination Theory (SDT) (Deci and Ryan, 2000), comprised four subscales: IM, IR, ER, and AM. Each subscale contained four items. Sample items included: for IM, “Because I think this activity is interesting”; for IR, “Because I think that this activity is good for me”; for ER, “Because I am supposed to do it”; and for AM, “I do this activity but I am not sure if it is worth it.” Participants completed the survey immediately after each aerobic dance session. The mean scores for each subscale were calculated and used as the primary outcomes.

#### Physical activity levels

The Yamax Digi-Walker SW-701 is a reliable pedometer for measuring PA levels in field settings. It records step counts, calculates distance based on individual stride length, and estimates caloric expenditure using total body weight. For this study, only step-count data were utilized. Prior to data collection, the pedometers were validated following the procedure recommended by Vincent and Pangrazi (2002). This involved shaking each pedometer vertically 100 times and comparing the recorded step count to the actual number of shakes. All pedometers showed a deviation of less than 5%, confirming their accuracy in step counting. Step data were expressed as steps per minute (SPM), calculated by dividing the total steps taken during the exercise session by the session’s duration (Scruggs et al., 2005). Participants were instructed to reset the pedometer to zero before the warm-up and return it at the end of each session.

### Data analysis

All data analyses were performed using SPSS version 27.0 (SPSS Inc., Chicago, IL, USA). Before conducting the primary analyses, descriptive statistics were computed to summarize participants’ demographic and session-related data. For the primary analyses, a repeated measures multivariate analysis of variance (MANOVA) was conducted to assess differences in enjoyment, situational motivation, self-efficacy, and PA steps between the two dance sessions (i.e., exergaming aerobic dance and traditional instructor-led aerobic dance), with Wilks’ Lambda testing differences between group means across the study variables. Group (exergaming aerobic dance vs. traditional aerobic dance) served as the within-subject factor for these comparisons. Statistical significance for all tests was set at *p* < 0.05. Additionally, the eta-squared effect sizes (η^2^) were calculated for each analysis to evaluate the magnitude of the observed effects. This quantifies the proportion of variance explained in the study variables by variation in the independent variables. Effect size values were interpreted based on established benchmarks, with 0.01 indicating a small effect, 0.06 a medium effect, and 0.14 or greater representing a large effect (Ryan and Deci, 2000). These analyses ensured a comprehensive understanding of the differences in participant responses between the two dance modalities.

## Results

Table 1 presents the full demographic and baseline anthropometric information for all participants. The final sample consisted of 40 young adults, including 20 females, with a mean age of 20.4 years (SD = 0.74), a mean height of 166.5 cm (SD = 8.5), and a mean weight of 58.7 kg (SD = 8.6). Only 5% of participants (2 out of 40) reported having prior experience with *Just Dance*. Given this small percentage, the potential for inaccurate RPE reporting due to familiarity with the exergaming platform was minimized. Additionally, most participants were of Han Chinese ethnicity (*n* = 32, 80%), with the remaining 20% comprising other ethnic groups.

**Table 1:** Demographic characteristics of participants.

Characteristics	*N*	Percentage
Previous just dance experience (n some: n none)	2	5%
	*Mean*	*SD*
Age (years)	20.4	0.74
Height (cm)	166.5	8.5
Weight (kg)	58.7	8.6
BMI (kg/m^2^)	21.1	0.34
*Ethnicity*	*N*	%
Han	32	80
Dong	5	12.5
Tujia	2	5
Zhuang	1	2.5
*Sex*		
Male	20	50
Female	20	50

BMI, body mass index.

**Figure 1:**
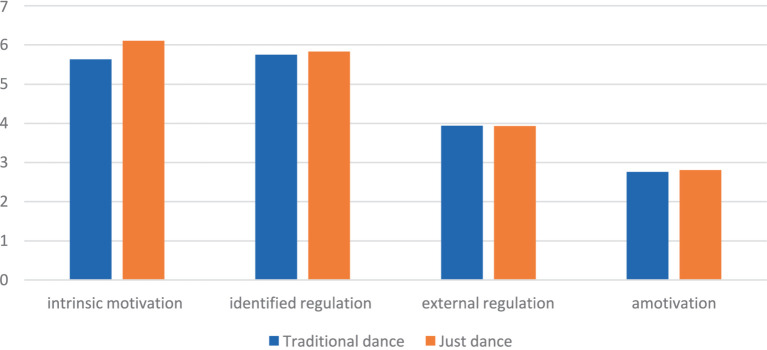
Comparison of means for situational motivation between exergaming dance and traditional aerobic dance.

The results indicate a significant main effect among two sessions for all study variables [Wilks Lambda = 0.44, *F* (1,33) = 6.07, *p* < 0.01, η^2^ = 0.56]. When comparing participants’ enjoyment between exergaming aerobic dance and traditional aerobic dance, participants had higher enjoyment in exergaming aerobic dance (*M* = 3.54, SD = 0.56) than in traditional aerobic dance (*M* = 3.31, SD = 0.60) with a small effect size [(η^2^ = 0.07), *F* (1,39) = 3.59, *p* = 0.05]. Also, Figure 1 shows that among the four situational motivation components, exergaming aerobic dance showed significantly higher IM (6.11 ± 0.98), (5.63 ± 1.31) compared to traditional aerobic dance (5.63 ± 1.31), *F* (1,39) = 3.83, *p* < 0.05, with a small effect being found (η^2^ = 0.09). No other significant differences were observed for other motivation outcomes.

There were no significant differences between exergaming dance (3.98 ± 0.60) and traditional aerobic dance (3.94 ± 0.85, η^2^ = 0.002) for self-efficacy. However, participants achieved significantly more steps per minute in the traditional aerobic dance (*M* = 136.39, SD = 9.83) compared to the exergaming session (*M* = 118.20, SD = 17.39), *F* (1,39) = 39.79, *p* < 0.01, η^2^ = 0.51 (see Table 2).

**Table 2:** Descriptive statistics for the outcome variables.

Variable	Traditional dance		Just dance		*p* value	Effect size (η^2^)
	Mean	SD	Mean	SD		
Enjoyment	3.31	0.60	3.54	0.56	0.05^b^	0.07
IM	5.63	1.31	6.11	0.98	0.05^b^	0.09
IR	5.75	0.90	5.83	0.83	> 0.05	0.01
ER	3.94	1.16	3.93	1.01	> 0.05	0.00
AM	2.76	1.14	2.81	1.23	> 0.05	0.001
Self-efficacy	3.94	0.85	3.98	0.60	> 0.05	0.002
Steps	136.39	9.83	118.20	17.39	<0.01^a^	0.51

IM, intrinsic motivation; IR, identified regulation; ER, external regulation; AM, amotivation; SD, standard deviation.

a Significantly less than traditional dance (*p* < 0.01).

b Significantly less than exergaming Just Dance (*p* < 0.05).

## Discussion

The present study compared differences in enjoyment, situational motivation, self-efficacy, and PA while practicing exergaming dance and traditional aerobic dance among Chinese young adults. Findings suggest that exergaming dance elicited significantly higher enjoyment and IM compared to traditional aerobic dance, whereas participants had significantly lower step count while using a popular exergaming dance application (Just Dance). The findings showed no significant differences in individuals’ self-efficacy between exergaming dance and traditional aerobic dance.

We hypothesized that college students would experience higher enjoyment, IM, IR, and self-efficacy in exergaming dance than in aerobic dance. Our findings partially supported this hypothesis, showing that college students reported greater enjoyment during exergaming dance sessions compared to traditional dance, suggesting exergaming may be more appealing and enjoyable than traditional aerobic exercise. Participants demonstrated higher levels of intrinsic IM during the exergaming dance session compared to the traditional dance session. Research consistently links IM to positive outcomes, such as increased adherence to PA and positive affect (Ryan and Deci, 2000). The enjoyment derived from exergaming dance may encourage participants to engage in the activity more frequently, which could play a crucial role in maintaining PA. The importance of IM in promoting PA participation is well-documented (Vallerand, 2007; Teixeira et al., 2012). A study by Liu et al. (2019) found that college students demonstrated higher IM when using virtual reality (VR) bikes compared to traditional bike sessions. Similarly, exergaming dance may foster greater IM by offering a more enjoyable and engaging experience than traditional dance, which could lead to increased PA participation and reduced sedentary behaviors. IM is typically associated with the enjoyment or pleasure derived from an activity, meaning participants in exergaming dance may feel more intrinsically motivated because they find the activity fun and entertaining. These findings suggest that exergaming dance could be an effective tool for enhancing IM and promoting PA participation among college students.

The lack of significant differences in IR between exergaming dance and traditional aerobic dance could be attributed to the goal-oriented nature of these activities. IR implies engaging in activities because they align with personal goals and values, which may apply to both exercise modalities to a similar extent for the participants (Liu et al., 2019). Participants may view both types of dance exercises as relatively equal means of improving physical fitness and overall health, thus leading to comparable levels of IR. Additionally, the structured format of both experiment sessions, having clear objectives and guided routines, might have provided similar experiences that each seem to support participants’ sense of purpose and align with personal goals. The similarity in perceived benefits and structured delivery could explain why IR did not differ significantly between the two modalities. In our findings, ER and AM showed the lowest average score in both groups when compared to other motivation categories which suggests that participants are motivated by inherent satisfaction. This echoes the finding of a previous study (Hwang et al., 2023) which found no significant difference between EM and AM in both the exergaming intervention and the comparison group.

Our findings revealed that young adults exhibited similar levels of self-efficacy in exergaming dance and traditional aerobic dance. This result does not support our second hypothesis but aligns with findings from a recent study involving another sample of Chinese college students (Gu et al., 2023). A plausible explanation is that self-efficacy for exergaming and traditional aerobic exercise is comparable because both activities demand similar levels of physical and mental effort for success. The physical exertion involved in exergaming mirrors that of traditional aerobic exercise, as both require intense movement and significant endurance (McDonough et al., 2020; Liu et al., 2019; McDonough et al., 2018). Additionally, both activities necessitate mental focus and concentration to achieve the desired outcomes (McDonough et al., 2020; da Silva et al., 2022; Meulenberg et al., 2022) and promoting comparable physical health benefits, including increased heart rate, improved cardiovascular health, and enhanced muscular strength and endurance. In addition, one brief exposure to the exergaming condition might not be enough to differentiate confidence levels, therefore a long-term intervention might unravel the possibility of differences in self-efficacy when exergaming dance condition is compared with traditional aerobic dance condition. Overall, the similarity in the physical and mental demands of these activities likely explains why young adults report equivalent self-efficacy levels for exergaming dance and traditional aerobic exercise.

Our findings also showed that college students took significantly fewer steps during exergaming dance sessions compared to traditional aerobic dance. Research suggests that individuals may engage differently depending on the mode of instruction. For example, participants may not have executed movements as vigorously or with full range of motion when following a virtual figure in the gaming situation compared to an in-person instructor who provided role model and visual prompts. The difference highlights the importance of delivery method in shaping participant engagement and exertion during PA interventions. This finding contributes to the growing body of evidence assessing whether exergaming can effectively promote PA among young adults. Exergaming is often compared to traditional aerobic exercise because it offers many similar benefits while also presenting unique advantages (Çakir-Atabek et al., 2020; Ferreira et al., 2022; Hwang et al., 2019; Park et al., 2020). Like traditional aerobic exercise, exergaming requires physical engagement through movements such as hopping, jumping, or other dynamic actions (Sousa et al., 2022; Pasco and Roure, 2022; McDonough et al., 2018; Oppici et al., 2022). This physical engagement serves as a form of exercise and has been shown in some cases to promote PA, making exergaming a potential alternative for encouraging active lifestyles.

The findings support our hypothesis, indicating that exergaming may be more appealing and enjoyable than traditional aerobic exercise. This aligns with prior research comparing aerobic exercise modalities in adult populations (Gao et al., 2014; Gao et al., 2013; Gao, 2012). The mode of exercise appears to significantly influence perceived enjoyment. For example, in a study by Bartlett et al. (2011) interval-based aerobic exercises, such as exergaming, resulted in significantly higher enjoyment than continuous, steady-state aerobic exercises like treadmill walking. This may be attributed to the dynamic and engaging nature of interval exercises, which contrasts with the monotonous nature of steady-state exercises, enhancing both exercise reinforcement and enjoyment (Bartlett et al., 2011). Adults often find exergaming more enjoyable because it incorporates elements of fun and competition (McDonough et al., 2018; Morais et al., 2022). By blending the physical benefits of traditional aerobic exercise with the interactivity and engagement of video games, exergaming offers an immersive and enjoyable experience.

Though, research on exergaming-related enjoyment among college students is limited and shows mixed findings. Garn et al. (2012) found that only obese participants preferred exergaming over traditional treadmill walking, while Barkley et al. (2016) reported no BMI-related differences in enjoyment when comparing exergaming to treadmill walking. However, Exergaming promotes exploration and experimentation, providing a diverse range of activities across various games. Its engaging nature increases the likelihood that individuals will commit to their fitness goals and enjoy the process (McDonough et al., 2018; Seo et al., 2023). Features such as visually appealing graphics and rewards for achieving goals further boost motivation, making exergaming more enjoyable than traditional aerobic exercise and encouraging longer, more consistent workout sessions (Liu et al., 2019; McDonough et al., 2018). In summary, exergaming delivers benefits comparable to traditional aerobic exercise while offering additional features that enhance motivation and enjoyment, establishing it as a valuable alternative for promoting PA.

Compared to traditional aerobic exercise, exergaming dance involves fewer activity steps but provides greater enjoyment and IM for PA. As an innovative exercise option, it offers a refreshing alternative to the repetitive nature of conventional workouts. Exergaming dance presents an effective way for young adults to stay active, maintain fitness, and enjoy the process. This study is distinctive in its examination of two dance modalities—exergaming dance and traditional aerobic dance—focusing on their effects on enjoyment, situational motivation, self-efficacy, and activity steps among Chinese college students. However, several limitations should be acknowledged. The study did not analyze differences between male and female participants. Future research should recruit larger samples to analyze potential gender differences in response to dance programs. It is worthy of note that the study’s repeated-measure design limits its ability to establish causal relationships, which should necessitate a Randomized Control Trial study to establish causality in future studies. Additionally, the study was conducted at a single university in China, which may limit the generalizability of the findings to other age groups or cultural contexts. The session duration was restricted to 12 min, capturing only acute responses; longer sessions may produce different outcomes. Furthermore, the dance routines differed between the two sessions, which may have influenced the outcomes. Future research should consider using closely matched or identical dance routines across conditions to ensure that the only variable being tested is the delivery method (i.e., exergaming vs. instructor-led). This approach would enhance the study’s internal validity by isolating the medium as the primary factor influencing results. Finally, physiological indicators such as heart rate and caloric expenditure were not assessed. As such, while step count serves as a useful proxy for physical activity volume, it does not fully reflect exercise intensity. Future research should employ experimental designs with more robust methodologies, such as a randomized controlled trial over several weeks to deepen understanding of the effects of exergaming dance.

## Conclusion

The findings highlight that exergaming dance significantly enhances perceived enjoyment and IM, making it an appealing alternative to traditional exercise. However, it was observed that exergaming dance sessions resulted in fewer steps per minute compared to instructor-led aerobic dance, suggesting a trade-off between physical intensity and engagement. These results have practical implications for designing PA interventions. Exergaming dance’s ability to foster enjoyment and reduce psychological barriers like boredom or low motivation makes it an effective way to encourage individuals, particularly those less inclined toward traditional workouts, to adopt and sustain an active lifestyle. By integrating gamified elements, exergaming can promote long-term adherence to PA, especially in populations like young adults or sedentary individuals.

## Data Availability

The raw data supporting the conclusions of this article will be made available by the authors, without undue reservation.
